# Caffeic Acid Phenethyl Ester: Consequences of Its Hydrophobicity in the Oxidative Functions and Cytokine Release by Leukocytes

**DOI:** 10.1155/2014/793629

**Published:** 2014-08-28

**Authors:** Luana Chiquetto Paracatu, Carolina Maria Quinello Gomes Faria, Camila Quinello, Camila Rennó, Patricia Palmeira, Maria Luiza Zeraik, Luiz Marcos da Fonseca, Valdecir Farias Ximenes

**Affiliations:** ^1^Department of Clinical Analysis, School of Pharmaceutical Sciences, São Paulo State University (UNESP), 14801-902 Araraquara, SP, Brazil; ^2^Department of Pediatrics, Medical School, University of São Paulo, 05403-900 São Paulo, SP, Brazil; ^3^Department of Organic Chemistry, Institute of Chemistry, São Paulo State University (UNESP), 14800-900 Araraquara, SP, Brazil; ^4^Department of Chemistry, Faculty of Sciences, São Paulo State University (UNESP), 17033-360 Bauru, SP, Brazil

## Abstract

Numerous anti-inflammatory properties have been attributed to caffeic acid phenethyl ester (CAPE), an active component of propolis. NADPH oxidases are multienzymatic complexes involved in many inflammatory diseases. Here, we studied the importance of the CAPE hydrophobicity on cell-free antioxidant capacity, inhibition of the NADPH oxidase and hypochlorous acid production, and release of TNF-α and IL-10 by activated leukocytes. The comparison was made with the related, but less hydrophobic, caffeic and chlorogenic acids. Cell-free studies such as superoxide anion scavenging assay, triene degradation, and anodic peak potential (*E*_pa_) measurements showed that the alterations in the hydrophobicity did not provoke significant changes in the oxidation potential and antiradical potency of the tested compounds. However, only CAPE was able to inhibit the production of superoxide anion by activated leukocytes. The inhibition of the NADPH oxidase resulted in the blockage of production of hypochlorous acid. Similarly, CAPE was the more effective inhibitor of the release of TNF-α and IL-10 by *Staphylococcus aureus* stimulated cells. In conclusion, the presence of the catechol moiety and the higher hydrophobicity were essential for the biological effects. Considering the involvement of NADPH oxidases in the genesis and progression of inflammatory diseases, CAPE should be considered as a promising anti-inflammatory drug.

## 1. Introduction

Propolis is a resin produced by honeybees and its chemical composition, colour, and aroma are changed according to geographical zones. Despite the chemical composition diversity, phenolic compounds are constituents that are always present in this natural product [1]. Among them, caffeic acid phenethyl ester is one of the propolis active components for which many potentially beneficial health properties have been demonstrated. Some recent findings include its antithrombotic potential through the inhibition of tumour necrosis factor- (TNF-)α induced endothelial tissue factor expression and activity [2]. Suppression of the phosphoinositide 3-kinase/AKT/XIAP pathway has also been shown to lead to apoptosis in melanoma tumour cells both* in vitro* and* in vivo* [3]. Application is used for the treatment of burn wound healing, leading to a decrease in inflammatory parameters and in oxidative damage [4] and also has anti-*Helicobacter pylori* activity, through the inhibition of the* Helicobacter pylori* peptide deformylase [5].

NADPH oxidases are multienzymatic complexes which catalyse the one-electron reduction of molecular oxygen to superoxide anion radical (O_2_^•−^) and are expressed in a variety of cell types. This multicomponent enzyme system is composed of two transmembrane proteins, p22phox and gp91phox, three cytosolic proteins, p47phox, p67phox, and p40phox, and a small G-protein, Rac [6]. The activation of NADPH oxidase involves the migration of the cytosolic proteins to the membrane, assembly of the enzyme complex, and the release of O_2_^•−^ into the intraphagosomal or extracellular space [7]. From O_2_^•−^, a cascade of enzymatic reactions takes place, leading to the production of hydrogen peroxide (H_2_O_2_), hydroxyl radical (^•^OH), and hypochlorous acid (HOCl) [7].

Besides its essential role in the innate immune defence, there is increasing evidence of the involvement of NADPH oxidases in the genesis and progression of vascular, inflammatory, and degenerative diseases [8–12]. Hence, inhibitors of NADPH oxidases represent an alternative and promising therapeutic pathway for the treatment of these chronic inflammatory diseases [13]. Several phytochemicals have been proposed as potential inhibitors of NADPH oxidase, for instance quercetin [14], resveratrol [15], flavonoids [16], and apocynin [17]. In this scenario, we have recently demonstrated that the esterification of protocatechuic acid, a natural phenolic compound found in many edible and medicinal plants, significantly increased its efficacy as an inhibitor of the release of oxidants by stimulated neutrophils [18]. Compared to apocynin, which is the most employed inhibitor of NADPH oxidase, the heptyl ester of protocatechuic acid was about ten-fold more potent [18].

In light of these findings, here, we aim to study and compare caffeic acid and the related compounds, chlorogenic acid, caffeic acid phenethyl ester, and phenethyl cinnamate as potential inhibitors of NADPH oxidase enzymatic activity and cytokine production by leukocytes. The results confirmed our hypothesis, since a direct relationship was found between the hydrophobicity of the tested compounds and the cellular functions evaluated.

## 2. Materials and Methods

### 2.1. Chemicals

Caffeic acid phenethyl ester, caffeic acid, chlorogenic acid, phenethyl cinnamate, apocynin, 2,2′-azobis(2-amidinopropane) hydrochloride (AAPH), 2,4,6-Tri(2-pyridyl)-*S*-triazine (TPTZ), dimethyl sulphoxide (DMSO), Brij 35, tung oil, 5-fluortryptamine, 2,2-diphenyl-1-picrylhydrazyl (DPPH), 3,3′,5,5′-tetramethylbenzidine (TMB), lucigenin, phorbol 12-myristate 13-acetate (PMA), taurine, Histopaque-1077, and Histopaque-1119 were purchased from Sigma-Aldrich Chemical Co. (St. Louis, MO, USA). 2-(4-iodophenyl)-3-(4-nitrophenyl)-5-(2,4-disulfophenyl)-2H-tetrazolium monosodium salt (WST-1) was purchased from Santa Cruz Biotechnology (Santa Cruz, CA, USA). Amplex Red (10-acetyl-3,7-dihydroxyphenoxazine) was purchased from Invitrogen (Eugene, OR, USA). Myeloperoxidase (MPO) (EC 1.11.1.7) was purchased from Planta Natural Products (Vienna, Austria) and its concentration was determined from its absorption at 430 nm (*ε*_430_ = 89,000 M^−1 ^cm^−1^). Hydrogen peroxide was prepared by diluting a 30% stock solution and calculating its concentration from its absorption at 240 nm (*ε*_240_ = 43.6 M^−1 ^cm^−1^). All reagents used for buffers and mobile phases were of analytical grade. Stock solutions of the tested compounds were prepared in DMSO for cellular studies or in ethanol for electrochemical and DPPH scavenging assays. For the antioxidant assays, the DMSO stock solutions were diluted in 10 mM phosphate buffered saline pH 7.4 (PBS) generating working solutions of lower concentrations. Ultrapure Milli-Q water from Millipore (Belford, MA, USA) was used for the preparation of buffers and solutions. PMA stock solutions were prepared in DMSO at a concentration of 50.0 *μ*M and were diluted to 0.5 *μ*M in PBS at the time of use. TMB solution was prepared by dissolving 14 mM TMB and 100 *μ*M potassium iodide in 50% dimethylformamide and 50% acetic acid (800 mM) (v/v). A working suspension of human serum opsonised zymosan was prepared as previously described at a final concentration of 10 mg/mL [19].

### 2.2. Hydrophobicity Index

The molecular hydrophobicities of caffeic acid and its derivatives were calculated based on their log *P* values (partitioning coefficient in n-octanol/water) based on Crippen's fragmentation method and were performed using ChemDraw software (ChemDraw Ultra 7.0.1, CambridgeSoft) [20].

### 2.3. Cyclic Voltammetry

Voltammetric studies were performed and the oxidation potentials, measured as anodic peak potential (*E*_pa_), were obtained using an Autolab PGSTAT 30 potentiostat/galvanostat (Eco-Chemie, Utrecht, Netherlands). Voltammetric curves were recorded at room temperature using a 3-electrode setup cell. The working electrode was a glassy carbon disk electrode (GC electrode, 3 mm diameter). The counter electrode was a platinum plate and the reference was an Ag/AgCl saturated KCl at 3 M electrode. The working electrode surface was carefully polished with 0.5 *μ*m alumina slurries before each experiment and was thoroughly rinsed with distilled water. A solution of sodium phosphate buffer 0.2 M (pH = 7) was used as a supporting electrolyte. The solutions were purged with nitrogen for 5 min before recording the voltammograms. The ethanolic solutions (5 mM) of the compounds were diluted in the electrochemical cell at final concentrations of 0.1 mM using the supporting electrolyte solution. The cyclic voltammograms were recorded at a potential scan rate of 5 mV s^−1^ [21].

### 2.4. DPPH Scavenging Assay

Caffeic acid and its derivatives were incubated for 30 min with 100 *μ*M DPPH in ethyl alcohol in the dark. The scavenging activity was evaluated spectrophotometrically at 517 nm using the absorbance of unreacted DPPH radical as a control and was calculated as [(Absorbance of control − absorbance of sample)/(absorbance of control)] × 100 [22].

### 2.5. Triene Degradation Assay

These studies were performed as previously described with modifications [23]. An emulsion was prepared by mixing 2.5 mg of tung oil (not stripped of tocopherols) in 25 mL of PBS containing 17 *μ*M Brij 35. The solution was vigorously vortexed to produce a homogeneous emulsion. The assays were performed as follows: the tung oil suspension (50 *μ*L) was incubated with freshly prepared 1 mM AAPH (source of peroxyl radical) in PBS at 37°C in the absence (control) or presence of the tested compounds in the wells of a microplate for 3 hours. The final reaction volume was 200 *μ*L. The microplate was read at 5 min intervals with shaking for 5 seconds before the measurements were taken. The absorbances were measured at 273 nm using a Synergy 2 Multimode microplate reader (BioTek, Winooski, VT, USA). The degradation of the eleostearic acid (conjugated triene) produced the absorbance versus time curve for which the area under the curve (AUC) was calculated. Curves of (AUC_sample_ − AUC_control_) against the concentration of each test compound were plotted and their slopes were used as analytical parameters. Trolox was used as a reference antioxidant. The ratio (slope_sample_/slope_trolox_), which is known as trolox equivalent antioxidant activity (TEAC), was used to assess the relative antioxidant efficacy of the test compounds.

### 2.6. Reactivity with Hydrogen Peroxide

The reactivity of caffeic acid and its derivatives with H_2_O_2_ was monitored amperometrically with a H_2_O_2_-selective electrode coupled to a Free Radical Analyzer (TRB 4100, World Precision Instruments, USA). The reaction mixtures (3 mL) were composed of 10 nM HRP, 250 *μ*M H_2_O_2_, and 250 *μ*M of the tested compounds in 0.1 M phosphate buffer, pH 7.4, at 25°C. The reactions were triggered by adding HRP [24].

### 2.7. Isolation of Human Leukocytes

Blood samples were obtained from healthy volunteers using heparin as an anticoagulant. Polymorphonuclear neutrophil (PMN) and peripheral blood mononuclear cells (PBMC) were isolated by centrifugation on a Histopaque-1077/1119 gradient at 700 ×g for 30 min at room temperature [25]. After isolation, the cells were resuspended in PBS supplemented with 1 mM calcium chloride, 0.5 mM magnesium chloride, and 1 mg/mL glucose (supplemented PBS). Experiments were performed in accordance with regulations of the Research Ethics Committee (21496413.8.0000.5426), Faculty of Pharmaceutical Sciences, Unesp, São Paulo, Brazil.

### 2.8. Superoxide Anion Production by Activated Leukocytes (Lucigenin-Dependent Chemiluminescence Assay)

PMN and PBMC (1 × 10^6^ cells/mL) were preincubated at 37°C in supplemented PBS with the test compound for 10 min. Next, lucigenin (10 *μ*M) and PMA (100 nM) or opsonised zymosan (1 mg/mL) were added and the light emission was measured for 30 min at 37°C using a plate luminometer (Centro Microplate Luminometer LB960, Berthold Technologies, Oak Ridge, TN, USA). The final assay volume was 250 *μ*L. The integrated light emission was used as an analytical parameter to measure the superoxide anion produced by the stimulated cells. The inhibitory potency was calculated using the light emission generated by the control, in which activated cells were incubated in the absence of the test compounds as [26].

### 2.9. Superoxide Anion Production by Activated Neutrophils (WST-1 Assay)

These studies were performed as previously described with modifications [27]. PMN (1.0 × 10^6^ cells/mL) were preincubated at 37°C in supplemented PBS with the test compounds for 10 min. Next, WST-1 (500 *μ*M) and PMA (100 nM) were added and the extracellular release of O_2_^•−^ was measured by the reduction of WST-1, monitoring the absorbance increase at 450 nm for 30 min at 37°C; this was performed using Synergy 2 Multimode microplate reader (BioTek, Winooski, VT, USA). The inhibitory potency was calculated using the absorbance of the control, in which the PMA-activated cells were incubated in the absence of the test compounds as a reference.

### 2.10. Superoxide Anion Production by Xanthine/Xanthine Oxidase (WST-1 Assay)

The test compounds were incubated at 37°C in PBS supplemented with 500 *μ*M WST-1 and 100 *μ*M xanthine. The reactions were initiated by the addition of 0.05 unit/mL xanthine oxidase and the reduction of WST-1 was assessed by monitoring the absorbance increase at 450 nm for 15 min at 37°C using Synergy 2 Multimode microplate reader (BioTek, Winooski, VT, USA).

### 2.11. Production of Hydrogen Peroxide by Activated Neutrophils

The analysis of the production of H_2_O_2_ and release into the extracellular medium was evaluated using the fluorescent probe Amplex Red, as previously described with modifications [28]. PMNs (5 × 10^5^ cells/mL) were preincubated at 37°C in supplemented PBS with the test compounds for 10 min. Next, Amplex Red (500 *μ*M) and PMA (100 nM) were added and the extracellular release of H_2_O_2_ was measured fluorimetrically at 530/590 nm for 30 min at 37°C using a Synergy 2 Multimode microplate reader (BioTek, Winooski, VT, USA). The inhibitory potency was calculated using the absorbance of the control, in which the PMA-activated cells were incubated in the absence of the tested compounds as a reference.

### 2.12. Production of Hypochlorous Acid by Activated Neutrophils and by H_2_O_2_/MPO

These studies were performed as described previously with modifications [29]. The neutrophils (1.0 × 10^6^ cells/mL) were preincubated at 37°C for 10 min in supplemented PBS containing 10 mM taurine and the test compounds. The cells were stimulated by the addition of PMA (100 nM) and were incubated for additional 30 min at 37°C. The reactions were stopped by the addition of catalase (20 *μ*g/mL) and were centrifuged at 6000 rpm. Then, 200 *μ*L was transferred into a 96-well plate and the accumulated taurine chloramine was measured by adding 50 *μ*L of TMB solution. The oxidised TMB was detected spectrophotometrically at 655 nm using Synergy 2 Multimode microplate reader (BioTek, Winooski, VT, USA). The amount of HOCl produced was calculated using a standard curve which was generated using pure HOCl and was submitted to the same analytical protocol.

For the cell-free experiments, the reactions were performed in a 96-well plate containing PBS, taurine (10 mM), pure MPO (50 nM), H_2_O_2_ (50 *μ*M), and various concentrations of the tested compounds. The reactions were triggered by the addition of H_2_O_2_ and were incubated at 37°C. After 30 min, the reactions were stopped by the addition of catalase (20 *μ*g/mL) and the accumulated taurine chloramine was measured as described above. The chlorination inhibitory potency was calculated using the control, in which the MPO/H_2_O_2_ was incubated in the absence of the test compounds as a reference.

### 2.13. Cytokines Production by Activated Peripheral Blood Mononuclear Cells

Peripheral blood mononuclear cells (1 × 10^6^ cells/mL per well) were cultured in RPMI-1640 (Gibco, Life Technologies, Foster City, CA, USA) medium added with fetal bovine serum (FBS) (Sigma, St. Louis, MO, USA) at 37°C in an atmosphere of 5% CO_2_, overnight. Cells were stimulated with* Staphylococcus aureus* (10 to 1 microorganism per cell) after 5 hours of incubation with the test compounds. After additional 18 hours of incubation the supernatants were stored at −80°C. TNF-α and IL-10 were quantified by enzyme-linked immunosorbent assay (ELISA) using BD OptEIA Human TNF ELISA Set (Cat. no. 555212) and BD OptEIA Human IL-10 ELISA Set (Cat. no. 555157), respectively, according to the manufacturer's instructions.

### 2.14. Statistical Analysis

Comparisons were performed using one-way ANOVA multiple comparisons among means, with the Turkey's post hoc test. Results were considered statistically significant when *P* < 0.05. The results were expressed as mean and SEM.

## 3. Results and Discussion

### 3.1. Structures and Hydrophobicity Indexes

**Figure 1:**
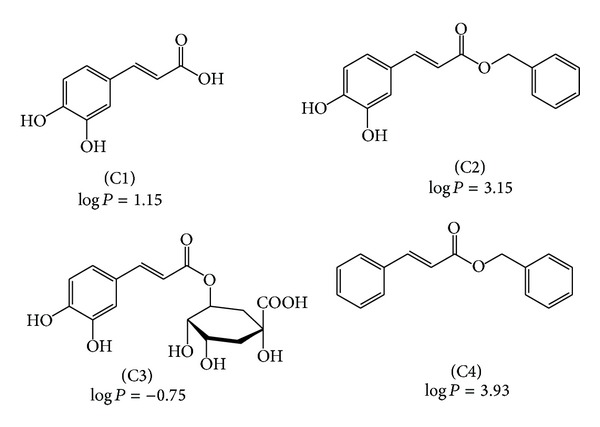
Molecular structures of caffeic acid, its derivatives, and hydrophobicity indexes (log *P*, partition coefficients n-octanol/water). Caffeic acid (C1), caffeic acid phenethyl ester (C2), chlorogenic acid (C3), and phenethyl cinnamate (C4).

As already known, the capacity of a substance to cross a lipid barrier may be crucial for its pharmacological effect [30]. Considering that NADPH oxidases are membrane-bound multienzymatic complexes, we hypothesised that, besides the redox properties, the hydrophobicity could also be relevant for the potency of NADPH oxidase inhibitors. This was our motivation for the study and comparison of caffeic acid and its derivatives as inhibitors of the production of oxidants by activated leukocytes.  Figure 1 shows the molecular structures and hydrophobicity indexes of caffeic acid (C1) and its derivatives, caffeic acid phenethyl ester (C2), chlorogenic acid (C3), and phenethyl cinnamate (C4). These compounds were selected with the aim of increasing or decreasing the hydrophobicity compared to C1, without any alteration in the oxidisable catechol moiety, C2, and C3, respectively, or by keeping the hydrophobicity and altering the oxidisability as in C4. From the partitioning coefficient n-octanol/water values depicted in Figure 1, it can be concluded that this goal was reached.

### 3.2. Oxidation Peak Potential

**Figure 2:**
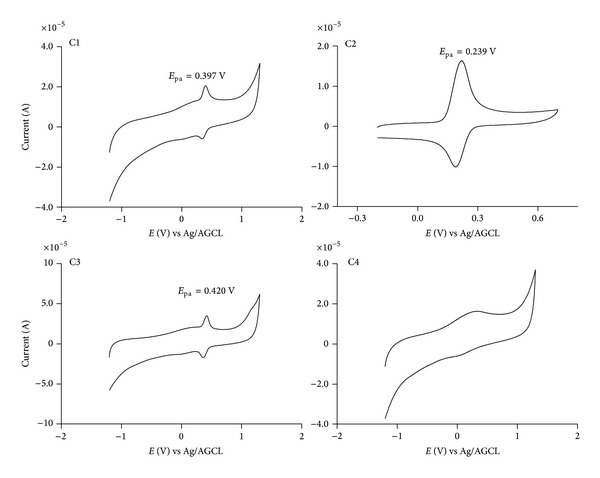
Cyclic voltammograms for caffeic acid and its derivatives (0.1 mM) obtained in 0.2 M sodium phosphate buffer at pH 7.0. The anodic peak potential (*E*_pa_) is indicated. The scan rate was 5 Mv*·*s^−1^.

Most of the substances that have been proposed as inhibitors of NADPH oxidase are redox active compounds, for example, the potent, but nonselective, and toxic diphenyleneiodonium [31]. This compound has a mechanism of action based on the generation of a transient radical and this inhibition occurs after direct phenylation of the redox active flavin prosthetic group of gp91phox, or of adjacent amino acid groups in the enzyme complex [31]. Another example is apocynin, which despite the same controversies about potency and selectivity, is the most commonly used NADPH oxidase inhibitor. For this molecule, the proposed mechanism of action is also dependent on its activation through MPO-mediated oxidation [6]. For this reason, prior to the cell-based studies, we measured and compared the oxidation potential of the tested compounds. Here, the oxidation peak potentials, which were measured as anodic peak potentials (*E*_pa_), were investigated by cyclic voltammetry using a glassy carbon working electrode. The cyclic voltammograms of the compounds obtained at pH 7.0 showed a well-defined anodic wave peaking in the range 0.24 to 0.42 V (Figure 2). It can also be observed that the esterification provoked only a small alteration in the oxidation peak potential, which was not unexpected, since esterification of carboxylic moiety does not provoke significant alterations in the oxidisability of these compounds. For instance, a comparison can be made with other phenolic acids, such as gallic acid (*E*_1/2_ 0.52 V) versus butyl gallate (*E*_1/2_ 0.51 V) [32] or protocatechuic acid (*E*_pa_ 0.222 V) versus heptyl protocatechuates (*E*_pa_ 0.266 V) [18]. For the compounds studied here, the exception was C4, which did not show a defined anodic wave in the applied voltage range. Obviously, this is a consequence of the absence of a phenolic moiety, making it a redox inactive compound.

### 3.3. Antioxidant Activity (Cell-Free Assays)

The cellular activation of the NADPH oxidase multienzymatic complex results in the generation of ROS; this phenomenon is usually monitored by the use of oxidisable luminescent, chromogenic, and fluorescent probes [33]. Hence, considering the importance of discrimination between the inhibition of the NADPH oxidase enzymatic activity and the simple and direct scavenging of ROS produced by the cells, we performed a complete panel of cell-free assays aiming to study the effects provoked by hydrophobicity on the antioxidant capacity of the test compounds.

Firstly, the compounds were studied by their efficacy as a reducing agent of the stable free radical DPPH. This assay is based on the reduction of this free radical, which is monitored by a decrease in the absorbance; the effective concentration (EC50) value expresses the concentration that is necessary to decrease the absorbance of DPPH by 50%. The EC50 values obtained were 15.7 ± 0.1, 16.4 ± 0.3, and 14.1 ± 0.1 *μ*M, for C1, C2, and C3, respectively, demonstrating that an alteration in the hydrophobicity did not cause a significant alteration in the reducing capacity of the DPPH free radical. However, as expected due to the absence of the catechol moiety, C4 was totally unable to reduce the DPPH free radical.

**Figure 3:**
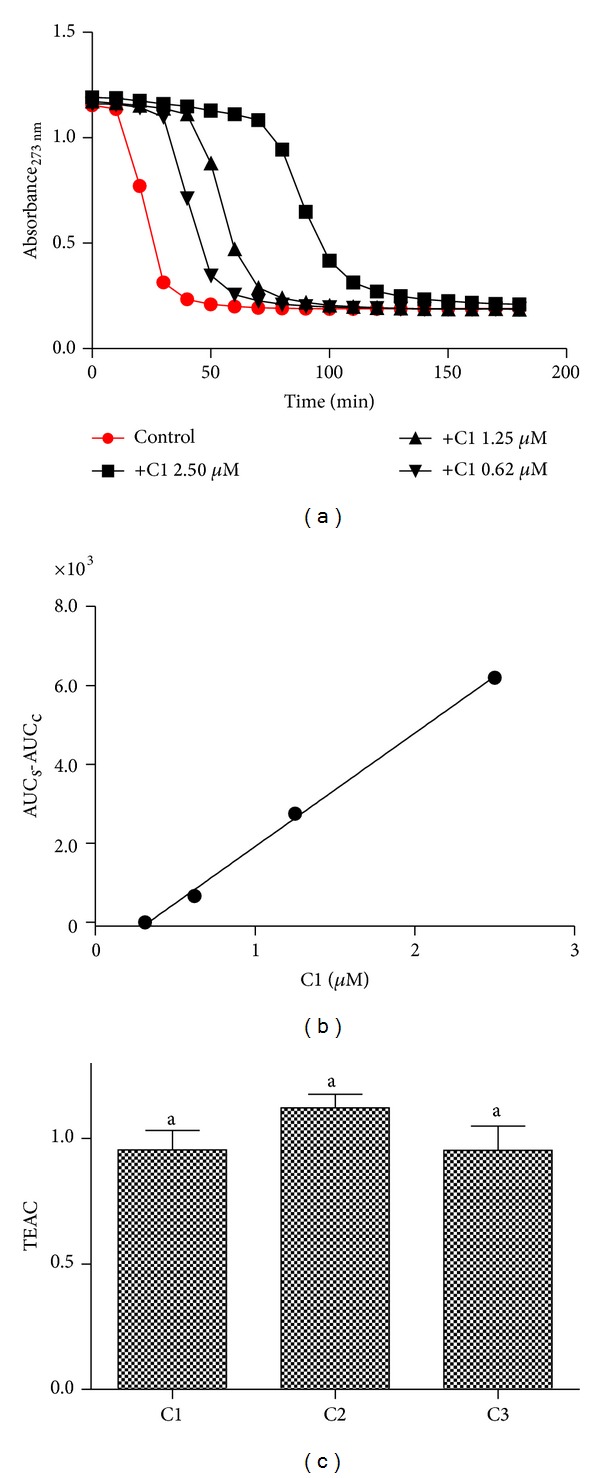
Inhibitory effect of caffeic acid and its derivatives on triene degradation by ROO^•^. (a) Bleaching of triene (eleostearic acid) by ROO^•^ and the effect provoked by the addition of C1. (b) Linear relationship between AUC and concentrations of C1. The slopes were calculated from the liner regression curves. (c) Trolox equivalent antioxidant activity (TEAC = slope compound/slope trolox). The results are mean and SEM of triplicate experiments. Different letters denote significant differences. One-way ANOVA and Tukey's multiple comparison test, *P* < 0.05.

In this sequence, the compounds were tested as inhibitors of the ROO^•^-mediated degradation of the conjugated triene present in eleostearic acid. In this assay the azocompound AAPH decomposes at 37°C in aqueous solutions to generate an alkyl radical (R^•^), which, in the presence of molecular oxygen, is converted to the corresponding peroxyl radical (ROO^•^). The oxidation of the triene was monitored by the bleaching of absorption at 273 nm. The addition of the tested compounds, by scavenging ROO^•^, delayed the bleaching and produced a concentration-dependent lag phase. The results depicted in Figure 3(a) show the kinetic profile of the fluorescence bleaching and the effect of the addition of C1 at increasing concentrations.  Figure 3(b) shows the relationship between the area under the curve and concentrations of C1 (similar results were obtained for C2 and C3, not shown). The slope of the linear regression (AUC/concentration) was used as an analytical parameter for assessment of the reactivity of the tested compounds with ROO^•^. The antioxidant trolox, a water soluble derivative of vitamin E, was used for comparison of the antiradical efficacy of the tested compounds. The ratio slope_sample_/slope_trolox_ generated the trolox equivalent antioxidant capacity (TEAC) values, which we used to measure the relative antioxidant efficacy of the test compounds. From the results depicted in Figure 3, it can be observed that the tested compounds were as effective as trolox, but no significant difference was obtained between them.

**Figure 4:**
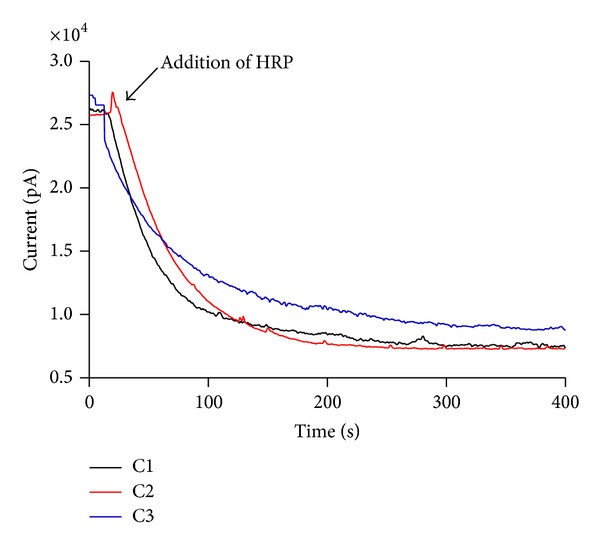
Reactivity of the test compounds with hydrogen peroxide. The reaction mixtures were comprised of 0.25 mM of the test compounds, 0.25 mM H_2_O_2_, and 10 nM HRP in 0.01 M PBS, pH 7.4, at 25°C. The results are representative of three experiments.

In the next step, caffeic acid and its derivatives were compared regarding their reactivity with H_2_O_2_. In these experiments, the reactions were monitored by amperometry using a H_2_O_2_ selective-electrode. The results displayed in Figure 4 show that the tested compounds were completely nonreactive with H_2_O_2_ in the absence of horseradish peroxidase (HRP). However, the addition of a catalytic amount of HRP caused an instantaneous consumption of H_2_O_2_. No significant difference was obtained between the tested compounds.

### 3.4. Inhibitory Effect on the Production of Superoxide Anion by Activated Leukocytes

As stated above, O_2_^•−^ is the initial ROS produced by the activation of NADPH oxidase. Hence, this reactive species was our first concern in the cellular assays. For that, caffeic acid and its derivatives were initially studied as inhibitors of lucigenin-dependent chemiluminescence elicited by activated neutrophils. In this reaction, O_2_^•−^ produced by the activation of the NADPH oxidase multienzymatic complex reduces lucigenin to form its cation radical, which reacts with a second O_2_^•−^ to form the energy-rich dioxetane molecule that decomposes, emitting light [26].

**Figure 5:**
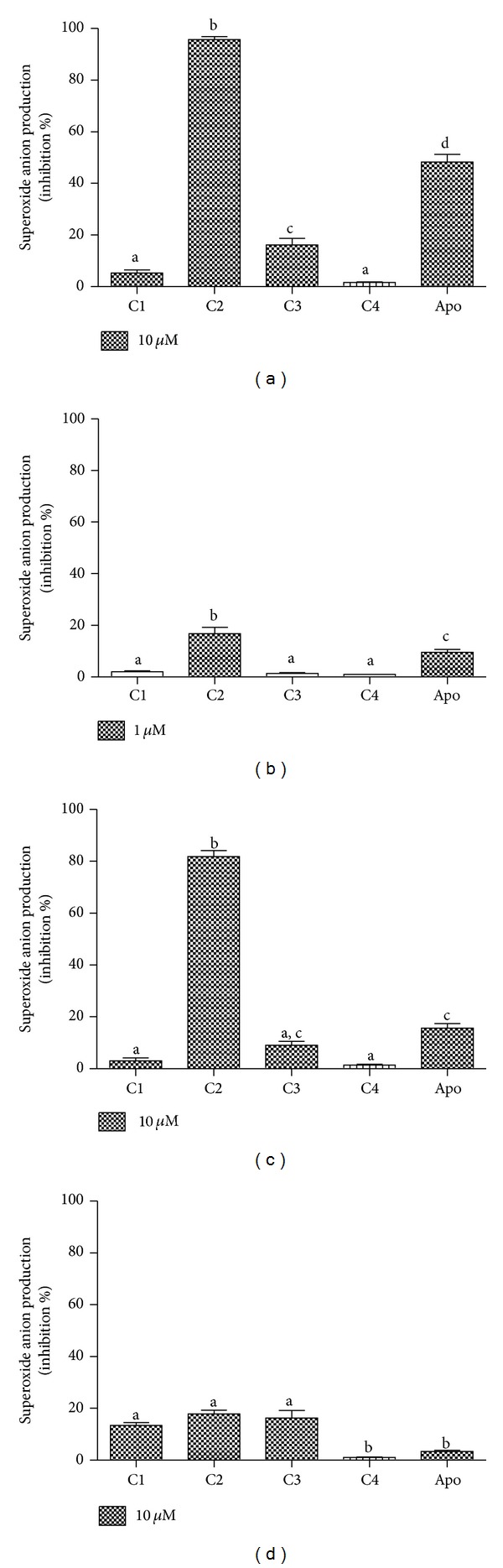
Caffeic acid, its derivatives, and apocynin (apo) as inhibitors of the production of superoxide anion by stimulated PMN and xanthine/xanthine oxidase. (a, b) Lucigenin-dependent chemiluminescence elicited by opsonised zymosan-activated PMN. (c) WST-1 reduction elicited by PMA-activated PMN. (d) WST-1 reduction elicited by xanthine/xanthine oxidase. The results are mean and SEM of duplicates of three different experiments. Different letters denote significant differences. One-way ANOVA and Tukey's multiple comparison test, *P* < 0.05.

In contrast to the previous antioxidant assays, C2 was found to be a significantly more potent inhibitor than the other compounds in this cell-based assay. As can be observed from the results depicted in Figures 5(a) and 5(b), C1 and C4 were totally ineffective, whereas C3 showed some activity, although this was much lower than that of C2.

The lucigenin-based assay is widely used for the detection of O_2_^•−^; however, some controversies have appeared regarding its selectivity for this reactive species [34]. For this reason and considering the higher efficacy of C2 compared to the other compounds, we also studied the activation of NADPH oxidase in neutrophils using a specific chromogenic probe to O_2_^•−^. This probe is the sulphonated tetrazolium salt (WST-1), which is similar to nitroblue tetrazolium (NBT), which is a widely used compound for the cytochemical determination of NADPH oxidase in leukocytes by microscopy. Compared to NBT, the advantage of WST-1 is its water solubility and membrane-impermeability. Hence, the formazan salt produced by its specific reaction with O_2_^•−^ can be detected in the extracellular medium using conventional absorbance measurements [27]. From the results depicted in Figure 5(c), we can conclude that the higher capacity of C2 was confirmed using the WST-1 assay. Indeed, the difference between C2 and the other compounds was still higher, which suggests its efficacy as an inhibitor of NADPH oxidase.

To confirm that C2 was indeed inhibiting the enzymatic activity of NADPH oxidase and not only acting as a scavenger of O_2_^•−^, an enzymatic and cell-free experiment was performed. For that, we used the xanthine/xanthine oxidase enzymatic system as a source of O_2_^•−^ (Figure 5(d)). As can be seen, using the same concentrations that were used in the cellular experimental model, the direct scavenger effects were minimal and, more importantly, the differences between C1, C2, and C3 were not statistically significant. It is worth noting that the inhibitory effects were not the result of a cytotoxic effect on leukocytes, as confirmed by the trypan blue exclusion assay. At the higher concentration used for the leukocyte studies, the viability of the cells was >98% (results not shown).

From the results depicted in Figure 5, it can be seen that apocynin was used as a reference inhibitor [35]. The reason for this choice was, obviously, the large applicability of apocynin as an NADPH oxidase inhibitor. Hence, the higher efficacy of C2 compared to apocynin is an additional indicator of its potential application as an anti-inflammatory compound, as has been widely demonstrated for apocynin.

**Figure 6:**
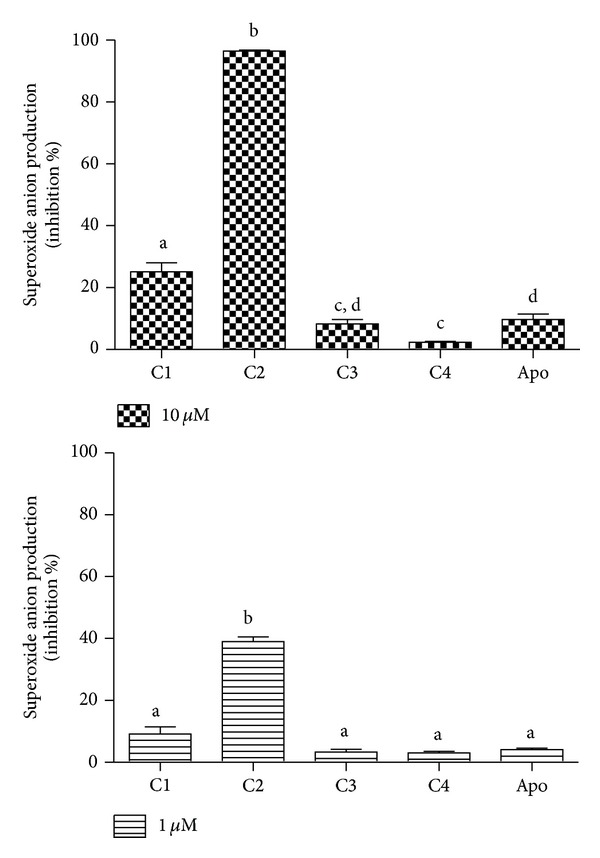
Caffeic acid, its derivatives, and apocynin (apo) as inhibitors of the production of superoxide anion by stimulated PBMC. Lucigenin-dependent chemiluminescence elicited by opsonised zymosan-activated PBMC. The results are mean and SEM of duplicates of three different experiments. Different letters denotes significant difference. One-way ANOVA and Tukey's multiple comparison test (*P* < 0.05).

The inhibitory potency of C2 was also observed using PBMC instead of neutrophils and again the more lipophilic ester was the more effective one (Figure 6). Our motivation for the use of PBMC relied on the fact that monocytes have a lower content of MPO compared to neutrophils. This is relevant, since MPO may play a role in the mechanism of NADPH oxidase inhibition. This is the case for apocynin, which must be oxidised to perform its role through an MPO-dependent reaction and the consequence is its lower efficacy as an NADPH oxidase inhibitor in PBMC [36]. From the results in Figure 6, it can be seen that the difference between C2 and apocynin was still higher using PBMC than neutrophils. This is an indication that C2 is not dependent on MPO for its biological effect.

### 3.5. Inhibitory Effect on the Production of Hydrogen Peroxide by Activated Leukocytes

**Figure 7:**
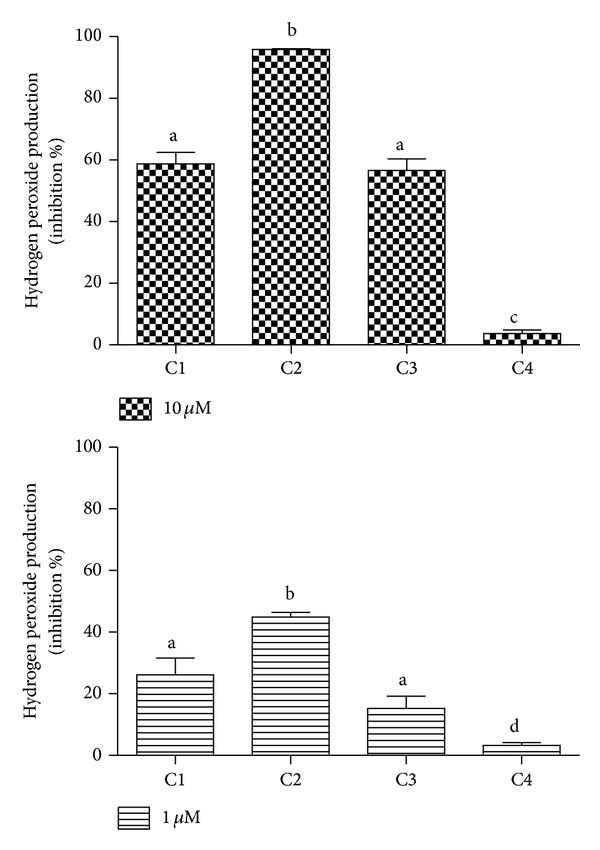
Caffeic acid and its derivatives as inhibitors of the production of hydrogen peroxide by stimulated PMN. The results are mean and SEM of duplicates of three different experiments. Different letters denote significant differences. One-way ANOVA and Tukey's multiple comparison test, *P* < 0.05.

In the enzymatic cascade for the production and release of oxidants through NADPH oxidase activation, the dismutation of O_2_^•−^ to H_2_O_2_ is the second step [7]. Hence, we also measured and compared the tested compounds as inhibitors of H_2_O_2_ release by activated neutrophils. The measurement of the production of H_2_O_2_ was performed using Ample Red, a specific fluorescent probe for this reactive species, in the extracellular medium [28]. From the data depicted in Figure 7, it can be concluded that C2 was still the more potent inhibitor. The detection of H_2_O_2_ by Amplex Red is based on its oxidation through a peroxidase-catalysed reaction (MPO in the case of neutrophils). Hence, it is obvious that any redox active compound, such as the phenolic acid derivatives used here, could compete with Amplex Red and provoke an inhibitory effect via the direct scavenging of H_2_O_2_. This fact could explain why, not only C2, as seen in the previous assay, but also C1 and C3, provoked an inhibitory effect in the production of H_2_O_2_. These results must be also analysed in light of the previous cell-free based assay for H_2_O_2_, where no difference was obtained between the studied compounds (Figure 4). Hence, the higher efficacy of C2 in this cell-based assay is one more confirmation of its effect on NADPH oxidase in leukocytes rather than this being a simple scavenging effect.

### 3.6. Inhibition of the Production of Hypochlorous Acid by Activated Leukocytes

**Figure 8:**
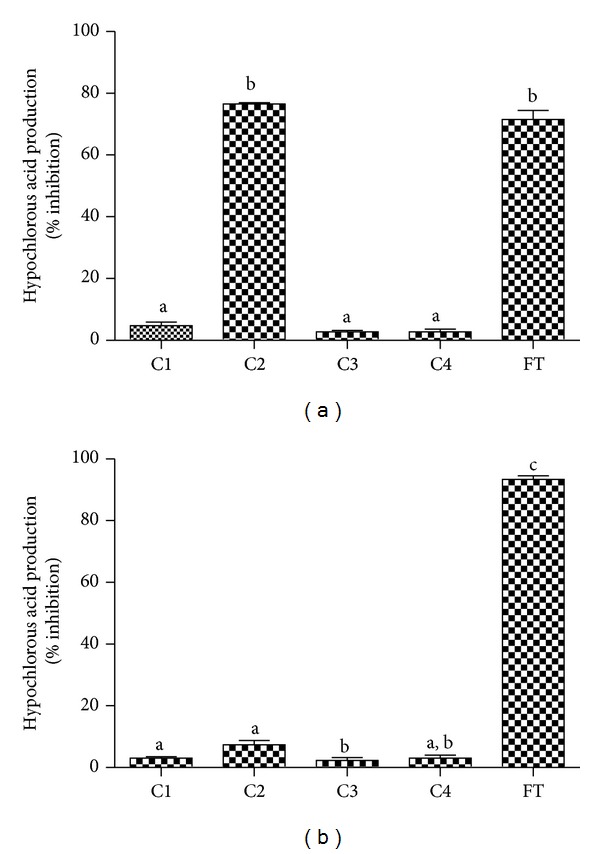
Caffeic acid, its derivatives, and 5-fluortryptamine (FT) as inhibitors of the production of hypochlorous acid by (a) stimulated PMN and (b) purified MPO. The results are mean and SEM of duplicates of three different experiments. Different letters denote significant differences. One-way ANOVA and Tukey's multiple comparison test, *P* < 0.05.

The next step in the enzymatic cascade of reactions that characterises the oxidative burst of neutrophils is the production of HOCl through the MPO-mediated oxidation of chloride using H_2_O_2_ as a cosubstrate. Hence, if C2 was indeed inhibiting the production of O_2_^•−^, the production of HOCl should also be affected. To study this possibility, we used a technique based on trapping the HOCl produced with taurine, which is converted to taurine chloramine. This stable oxidant was detected by the iodide-catalysed oxidation of TMB [37]. From the results depicted in Figure 8(a), it can be noted that, among the phenolic acid derivatives, only C2 was able to inhibit the production of HOCl.

Three different reasons could explain the decreased production of HOCl by activated neutrophils in the presence of C2: (i) inhibition of the enzymatic activity of MPO, (ii) direct scavenging of HOCl, and (iii) inhibition of the production of O_2_^•−^. To discriminate these effects, we measured the capacity of the tested compounds as inhibitors of the chlorinating activity of the purified MPO. From the results depicted in Figure 8(b), we can note that all of the tested compounds, including C2, were unable to inhibit the formation of HOCl in the cell-free system. These results also eliminated the direct scavenging effect upon HOCl. Hence, the inhibition of the production of HOCl without affecting the MPO enzymatic activity is an additional confirmation of the effect of C2 on NADPH oxidase in neutrophils.

In the HOCl experiments, we used 5-fluortryptamine as a reference inhibitor. This indole derivative is a potent inhibitor of the chlorinating activity of MPO [38]. In agreement with that, in our hands, 5-fluortryptamine was able to block the production of HOCl in both cell and cell-free assays (Figures 8(a) and 8(b)). However, C2 was only able to exert its effect on neutrophils, which reinforces its specific action upon the NADPH oxidase enzymatic system.

### 3.7. Inhibition of the Production of TNF-α and IL-10

Tumour necrosis factor-α (TNF-α) is a major proinflammatory cytokine involved in the inflammatory response. There is evidence of crosstalk between NADPH oxidase and TNF-α in many experimental models using several cell lineages. Some recent findings include the following: the inhibition of NADPH oxidase attenuated TNF-α impaired endothelium-dependent vasodilation [39], increased TNF-α expression associated with diabetes contributes to erectile dysfunction by promoting NAPDH oxidase-mediated ROS generation [40], and TNF-α produces oxidative stress in neuronal cells via the activation of NADPH oxidase through a ceramide-mediated mechanism [41] and so on. Moreover, TNF-α has been shown to prime the neutrophil respiratory burst through the partial phosphorylation of p47PHOX [42], which is a cytosolic component of the NADPH oxidase complex.

From the opposite point of view, oxidative stress, which can be initiated by the activation of NADPH oxidase, among other mechanisms, can promote the release of TNF-α through the activation of redox-sensitive transcription factor as nuclear factor-kappa B (NF-kappa B) [43, 44]. Hence, there are numerous demonstrations of the beneficial effect of natural compounds that, besides antioxidative effects, also decrease the production of TNF-α and other proinflammatory cytokines [10, 45, 46]. C2 is included in this class of natural antioxidants that are able to inhibit the release of TNF in many experimental models and using different cell/tissue models [2, 47, 48]. Indeed, C2 was described as a potent and a specific inhibitor of NF-kappa B in the human U937 cell lineage [49].

**Figure 9:**
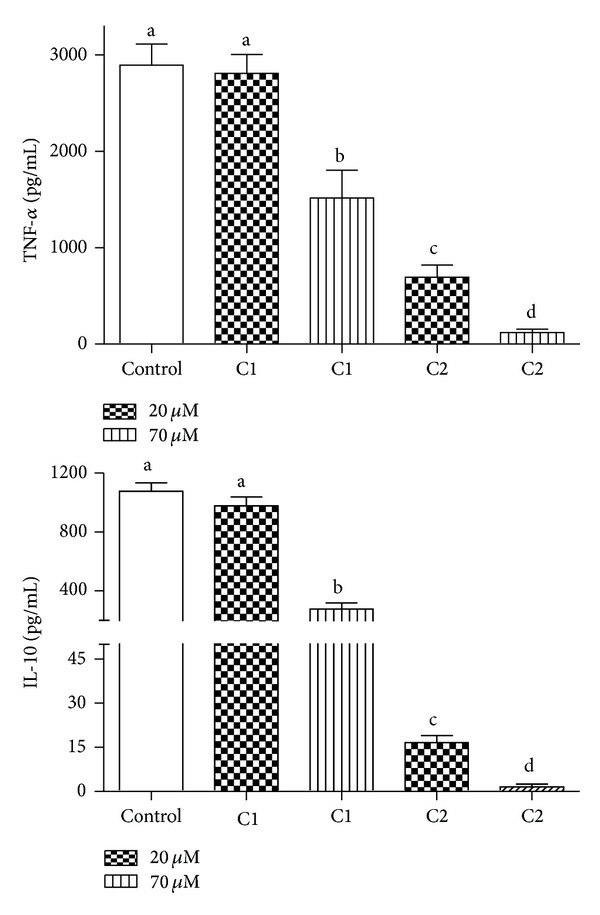
Production of TNF-α and IL-10 by stimulated PBMC and the inhibitory effect of caffeic acid (C1) and caffeic acid phenethyl ester (C2). The results are mean and SEM of duplicates of six different experiments. Different letters denote significant differences. One-way ANOVA and Tukey's multiple comparison test, *P* < 0.05.

Considering the relationship between NADPH oxidase and TNF-α production, we also compared C1 and C2 as inhibitors of the release of TNF-α by* Staphylococcus aureus* activated PBMC. From the results depicted in Figure 9, it can be concluded that the higher hydrophobicity of C2 was also relevant regarding the inhibition of TNF-α. As discussed above, it would be expected that C2 showed this inhibitory effect, but the finding that it was significantly more effective than C1, which has similar antioxidant potency, is additional evidence that its hydrophobicity is an important factor in this biological effect, which is closely related to NADPH oxidase activation.

Similar results were obtained for IL-10, an anti-inflammatory cytokine whose production by stimulated PBMC is also inhibited by natural antioxidants [50]. Regarding the relationship between NADPH oxidase and IL-10, it has been proposed that this cytokine down regulates the polymorphonuclear neutrophil production of ROS [51]. Hence, similarly to TNF-α, our finding that C2 was also more effective than C1 as an inhibitor of the production of IL-10 could also be a consequence of the relationship between NADPH oxidase and this cytokine.

## 4. Conclusions

Owing to the involvement of ROS in the pathophysiology of many diseases, several research groups are striving to develop an effective and nontoxic NADPH oxidase inhibitor, which is one of the most important primary sources of superoxide anion in the cells. Here, we have demonstrated, for the first time, that caffeic acid phenethyl ester, besides its extensively described potentially beneficial health properties, is also an effective inhibitor of NADPH oxidase. Our results clearly showed that, besides the redox active catechol moiety, the increased hydrophobicity provoked by the esterification was decisive for this activity. As demonstrated using a panel of assays, the cell-free antioxidant capacity of caffeic acid phenethyl ester was not significantly different from that of the more hydrophilic caffeic acid and chlorogenic acid. On the other hand, in the cell-based assays, caffeic acid phenethyl ester was always the more effective inhibitor. It seems clear that these results are a consequence of the capacity of caffeic acid phenethyl ester to diffuse into the cells and to reach the membrane-bound NADPH oxidase multienzymatic complexes.

As a potential NADPH oxidase inhibitor, this propolis component can be used for the treatment of chronic inflammatory diseases; hence, a remaining question is what dosage should be used to reach a pharmacologically efficient plasma concentration. In this context, an analytical method to determine caffeic acid phenethyl ester in rat plasma and urine was developed and the pharmacokinetic studies showed that it is rapidly absorbed and excreted in urine both as an unmodified molecule and as a glucuronide conjugate [52]. In this study, 100 mg/kg was orally administered, but the recovered caffeic acid phenethyl ester was only 0.007 to 0.021%, which is an indication that it was promptly hydrolysed by plasmatic esterases [52], probably to its caffeic acid precursor. As we have demonstrated here, caffeic acid was significantly less efficient compared to the ester derivative, hence an effective dose of caffeic acid phenethyl ester for* in vivo* studies could be significantly higher than that obtained in cell-based studies. For this reason, we suggest that the effective concentrations of caffeic acid phenethyl ester found in cell-based studies should be only used as an initial parameter for* in vivo* applications.
